# Intercomparison of methods to estimate gross primary production based on CO_2_ and COS flux measurements

**DOI:** 10.5194/bg-19-4067-2022

**Published:** 2022-09-01

**Authors:** Kukka-Maaria Kohonen, Roderick Dewar, Gianluca Tramontana, Aleksanteri Mauranen, Pasi Kolari, Linda M. J. Kooijmans, Dario Papale, Timo Vesala, Ivan Mammarella

**Affiliations:** 1 Institute for Atmospheric and Earth System Research/Physics, Faculty of Science, University of Helsinki, Helsinki, Finland; 2 Division of Plant Sciences, Research School of Biology, The Australian National University, Canberra, ACT 2601, Australia; 3 Image Processing Laboratory (IPL), Parc Científic Universitat de València, Universitat de València, Paterna, Spain; 4 Terrasystem s.r.l, Viterbo, Italy; 5 Meteorology and Air Quality, Wageningen University and Research, Wageningen, the Netherlands; 6 DIBAF, Department for Innovation in Biological, Agro-food and Forest Systems, University of Tuscia, Viterbo, Italy; 7 IAFES, Euro-Mediterranean Center for Climate Change (CMCC), Viterbo, Italy; 8 Institute for Atmospheric and Earth System Research/Forest Sciences, University of Helsinki, Helsinki, Finland

## Abstract

Separating the components of ecosystem-scale carbon exchange is crucial in order to develop better models and future predictions of the terrestrial carbon cycle. However, there are several uncertainties and unknowns related to current photosynthesis estimates. In this study, we evaluate four different methods for estimating photosynthesis at a boreal forest at the ecosystem scale, of which two are based on carbon dioxide (CO_2_) flux measurements and two on carbonyl sulfide (COS) flux measurements. The CO_2_-based methods use traditional flux partitioning and artificial neural networks to separate the net CO_2_ flux into respiration and photosynthesis. The COS-based methods make use of a unique 5-year COS flux data set and involve two different approaches to determine the leaf-scale relative uptake ratio of COS and CO_2_ (LRU), of which one (LRU_CAP_) was developed in this study. LRU_CAP_ was based on a previously tested stomatal optimization theory (CAP), while LRU_PAR_ was based on an empirical relation to measured radiation.

For the measurement period 2013–2017, the artificial neural network method gave a GPP estimate very close to that of traditional flux partitioning at all timescales. On average, the COS-based methods gave higher GPP estimates than the CO_2_-based estimates on daily (23% and 7% higher, using LRU_PAR_ and LRU_CAP_, respectively) and monthly scales (20% and 3% higher), as well as a higher cumulative sum over 3 months in all years (on average 25% and 3% higher). LRU_CAP_ was higher than LRU estimated from chamber measurements at high radiation, leading to underestimation of midday GPP relative to other GPP methods. In general, however, use of LRU_CAP_ gave closer agreement with CO_2_-based estimates of GPP than use of LRUPAR. When extended to other sites, LRU_CAP_ may be more robust than LRU_PAR_ because it is based on a physiological model whose parameters can be estimated from simple measurements or obtained from the literature. In contrast, the empirical radiation relation in LRU_PAR_ may be more site-specific. However, this requires further testing at other measurement sites.

## 1 Introduction

Photosynthetic carbon uptake (or gross primary production, GPP) is a key component of the global carbon cycle, with the terrestrial ecosystems removing approximately 30% of annual anthropogenic carbon dioxide (CO_2_) emissions from the atmosphere (Luo et al., 2015; Friedlingstein et al., 2020). With the current climatic warming it has been suggested that both photosynthesis and respiration are increasing due to the CO_2_ fertilization effect and rising temperatures providing more favourable conditions not only for photosynthesis but also for respiration (Dusenge et al., 2019). However, it is not known at which rate these two processes are changing and thus the extent to which they offset each other. In addition, their relative importance varies seasonally, with photosynthesis predicted to increase more than respiration in spring, leading to greater carbon uptake, while respiration is predicted to increase more than photosynthesis in autumn, leading to net carbon emission in northern terrestrial ecosystems (Piao et al., 2008). Methods to measure and study photosynthesis and respiration individually are thus crucial for future carbon cycle predictions.

Eddy covariance (EC) is widely used to measure the biosphere–atmosphere exchange of CO_2_ at the ecosystem scale. However, EC only measures net ecosystem CO_2_ flux (NEE), which includes contributions from both CO_2_ uptake by photosynthesis (GPP) and ecosystem respiration (*R*). Traditionally, NEE partitioning into GPP and respiration uses the method of Reichstein et al. (2005), in which temperature response curves are fitted to nighttime CO_2_ flux data (respiration). However, this method relies on nighttime EC flux measurements, which are uncertain and often filtered out due to low-turbulence conditions and possible advective gas transport (Aubinet, 2008). To address this problem, partitioning methods have been developed based on a combination of nighttime temperature responses of respiration (as in nighttime method) and daytime radiation responses of GPP (daytime method) (Lasslop et al., 2010; Kulmala et al., 2019). However, both the nighttime method and the daytime method assume that respiratory processes operate in the same way during the day and night and have uncertainties due to assumptions of functional relationships (Tramontana et al., 2020). These assumptions lead to uncertainties in partitioning because different biomass compartments (soil organic matter, roots, stems, branches, foliage) could have different drivers and respiration responses even within the same ecosystem (Kolari et al., 2009; Keenan et al., 2019). Leaf respiration during the day may be inhibited by radiation, the so-called Kok effect (Kok, 1949; Wohlfahrt et al., 2005; Heskel et al., 2013; Yin et al., 2020), and Keenan et al. (2019) and Wehr et al. (2016) suggest that, as a result, global GPP based on the nighttime method has been overestimated. On the other hand, photorespiration, which is an oxidation process competing with carboxylation under radiation, might offset inhibition by the Kok effect (Heskel et al., 2013).

One way to address these uncertainties in flux partitioning is to use machine learning methods, such as artificial neural networks, to separate NEE into respiration and GPP (Tramontana et al., 2020). The advantage of this method is that it makes no a priori assumptions about responses to environmental drivers but determines these based only on data. In a pioneering study, Desai et al. (2008) attempted to use an artificial neural network to emulate the nighttime partitioning method but obtained no significant improvements. More recently, Tramontana et al. (2020) proposed a new approach (NN_C-part_) involving novel methods for implementing the network’s structure and of inferring GPP and *R* signals from NEE. Both nighttime and daytime NEE are used for network training, so the dynamics of biophysical processes are accounted for in a comprehensive way.

Yet another approach to addressing uncertainties in GPP estimates is to use proxies for photosynthetic CO_2_ uptake. One such proxy is carbonyl sulfide (COS), which is a sulfur compound with a tropospheric mixing ratio of approximately 500 ppt (parts per trillion) (Montzka et al., 2007). While the use of different CO_2_-based partitioning methods is primarily aimed at more accurate GPP estimation, in contrast the use of COS as a proxy for GPP is aimed at a better process understanding of GPP. COS is mainly produced by oceans and anthropogenic sources (Kettle et al., 2002; Berry et al., 2013; Launois et al., 2015; Whelan et al., 2018), while vegetation is the largest sink (Sandoval-Soto et al., 2005; Blonquist et al., 2011). COS has been proposed as a proxy for GPP because it is taken up by plants through the same diffusive pathway as CO_2_ and transported to the chloroplast surface. There it is destroyed by a hydrolysis reaction catalysed by the enzyme carbonic anhydrase (CA, also located within the cytoplasm; Polishchuk, 2021), while CO_2_ continues its journey inside the chloroplast, where it is assimilated in the Calvin cycle (Wohlfahrt et al., 2012). It is assumed that COS is completely removed by hydrolysis so that there is no back-flux from the leaf to the atmosphere (Protoschill-Krebs et al., 1996). Estimates of GPP from COS flux measurements use the leaf relative uptake ratio (LRU), that is, the ratio of COS and CO_2_ deposition rates at the leaf scale. While LRU has been treated either as a global or plant-specific constant (Asaf et al., 2013; Stimler et al., 2012), recent studies have shown that LRU is a function of solar radiation because CO_2_ uptake is highly radiation dependent, while COS uptake is not (Stimler et al., 2010; Yang et al., 2018; Kooijmans et al., 2019; Spielmann et al., 2019) and may also vary with vapour pressure deficit (Sun et al., 2018b; Kooijmans et al., 2019). In addition to uncertainties related to variation in LRU, COS-based GPP estimates are uncertain because ecosystem-scale COS flux measurements typically have a low signal-to-noise ratio and high random uncertainty at a 30 min timescale, although this is reduced when fluxes are averaged over longer time periods (Kohonen et al., 2020).

In this study, we compare the annual, seasonal, daily, and sub-daily variation of (i) a traditional GPP estimate (GPP_NLR_, NLR referring to non-linear regression) based on a combination of daytime and nighttime methods, (ii) a neural network GPP estimate based on NEE and NN_C-part_ (GPP_ANN_), (iii) a GPP estimate based on COS flux measurements using the radiation-dependent LRU function from Kooijmans et al. (2019) (GPP_COS,PAR_), and (iv) a GPP estimate based on COS flux measurements using a previously published stomatal optimization model (CAP) to calculate LRU (GPP_COS,CAP_) in a boreal evergreen needle–leaf forest during the years 2013–2017. Our aim is to study potential inconsistencies in diel or seasonal patterns of GPP that may arise from extrapolating nighttime temperature responses to daytime ones, as well as to discuss the limitations and uncertainties of all four methods. We also make recommendations for improving COS-based GPP estimates.

## 2 Materials and methods

### 2.1 Site description

Measurements were conducted at the Hyytiälä forest Station for Measuring Ecosystem Atmosphere Relations (SMEAR) II measurement site (61°51′N, 24°17′E), where the forest stand is already more than 50 years old (Hari and Kulmala, 2005). The stand is dominated by Scots Pine *(Pinus Sylvestris* L.) with some Norway spruce *(Picea abies* L. Karst.) and deciduous trees (e.g. *Betula* sp., *Populus tremula, Sorbus aucuparia)*. The daytime flux footprint covers a ca. 50 ha area of the forest. The canopy height increased from approximately 18 to 20m during the measurement period (2013–2017), and the all-sided leaf area index (LAI) was ca. 8m^2^m^−2^.

### 2.2 Measurements

#### Eddy covariance fluxes and environmental measurements

EC measurements were made on a 23m high tower. The set-up consisted of a Gill HS (Gill Instruments Ltd., England, UK) sonic anemometer measuring horizontal and vertical wind velocities and sonic temperature, as well as a quantum cascade laser (QCL; Aerodyne Research Inc., Billerica, MA, USA) for measuring COS, CO_2_ and H_2_O mixing ratios at 10 Hz frequency. The set-up is described in more detail in Kohonen et al. (2020), and flux data are presented in Vesala et al. (2022). Flux processing was done using EddyUH software (Mammarella et al., 2016) following the methods presented by Kohonen et al. (2020). Fluxes were corrected for storage change and filtered according to friction velocity. Storage change fluxes of COS were calculated from the COS profile measurements in 2015–2017 and from concentration measurements at one height in other years, as described in Kohonen et al. (2020); CO_2_ storage change fluxes were calculated from CO_2_ concentration profile measurements. The friction velocity threshold was determined from CO_2_ fluxes (Papale et al., 2006), and a threshold of 0.3 ms^−1^ was applied to the entire data set to exclude periods of low turbulence. COS flux processing was done similarly to CO_2_ processing, but time lag and spectral corrections were determined from CO_2_ measurements and applied to COS as recommended by Kohonen et al. (2020). Gap-filling of the COS flux was done using empirical formulas based on photosynthetically active radiation (PAR) and vapour pressure deficit (VPD), as described by Kohonen et al. (2020). CO_2_ fluxes were gap-filled and partitioned using a procedure to be explained in more detail in Sect. 2.3.1.

Environmental measurements used in the study include air temperature (*T*_a_) at 16.8m (measured with a Pt100 temperature sensor inside a ventilated custom shield), PAR above the canopy (Li-190SZ quantum sensor, LI-COR, Lincoln, NE, USA), relative humidity (RH) at 16.8m height (Rotronic MP102H, Rotronic Instrument Corp., NY, USA), soil temperature (*T*_soil_) at 2–5 cm depth (KTY81-110 temperature sensor, Philips, the Netherlands) as a mean of five locations, and soil water content (SWC) in the humus layer (Delta-T ML2 soil moisture sensor, Delta-T Devices, Cambridge, UK).

### 2.3 GPP calculations

This section describes each of the four methods for estimating GPP. Daily average GPP was only calculated if more than 50% of the measured 30 min flux data were available for each day, and monthly averages were calculated from the daily means. In Vesala et al. (2022), COS fluxes were found to have 52% data availability on average. While setting a 50% threshold is somewhat subjective, it ensures that the analysed daily estimates of GPPs reflect measured fluxes rather than the gap-filling procedure. Gap-filled flux data were used in estimating diurnal variation and cumulative GPP. All comparisons between the methods used measured (non-gap-filled) data only, when both CO_2_ and COS flux data were available.

#### 2.3.1 GPP from traditional CO_2_ flux partitioning

NEE was partitioned into respiration (*R*) and GPP_NLR_ as

(1) $$\text{NEE}=R-{\text{GPP}}_{\text{NLR}},$$

where *R* was estimated as in the nighttime method,

(2) $$R=R_{\text{C}}Q_{10}^{T_{\text{sa}}/10},$$

where *R*_c_ is the respiration at a reference temperature (*T* = 0 °C), *Q*_10_ is the temperature sensitivity of *R*, and *T*_sa_ is the arithmetic mean between the air temperature at 16.8 m height and soil temperature at 5 cm depth. Previous studies have shown *T_sa_* to be a good choice of respiration driver at Hyytiälä forest (Kolari et al., 2009; Lasslop et al., 2012).

When NEE measurements were not available, the GPP model followed the formula

(3) $${\text{GPP}}_{\text{NLR}}=\left(\alpha \text{PAR}+P_{\max}-\sqrt{{\left(\alpha \text{ PAR }+P_{\max}\right)}^{2}-4\text{Θ}PARP_{\max}}\right)\frac{f\left(T_{\text{a}}\right)}{2\text{Θ}},$$

where *α*, *P*_max_, and Θ are fitting parameters, and *f* (*T*_a_) is an instantaneous temperature response that brings GPP gradually towards zero at freezing temperatures, given by

(4) $$f\left(T_{\text{a}}\right)=\frac{1}{1+e^{\left(2\left(T_{0}-T_{\text{a}}\right)\right)}},$$

where *T*_0_ = –2 °C is the inflection point (Kolari et al., 2014).

Parameters *α*, *P*_max_, and *R*_c_ were estimated for 15 d periods, while *Q*_10_ was estimated from the weighted mean of monthly *Q*_10_ values from June to August over several years. Weights were the inverse of the confidence interval of each *Q*_10_ estimate. Θ was determined as the value that gave the best model fit when the partitioning was run during summer months (June–August) over several years (Kulmala et al., 2019). The parameters of Eq. (3) were estimated from GPP partitioned with the nighttime method in Eq. (1). The modelled NEE from Eqs. (3) and (2) was compared with the measured NEE in Fig. B1a.

#### 2.3.2 GPP from artificial neural networks

GPP_ANN_ from the data-driven model was estimated by applying the NN_C-part_ algorithm (Tramontana et al., 2020). NN_C-part_ is a customized neural network that emulates the bio-physical processes driving both GPP and R at ecosystem scale and has been applied to several vegetation types distributed globally. The network consists of two subnetworks, which simulate GPP and *R*. The two subnetworks are connected in the last node of the overall structure, in which the GPP and R signals are combined to calculate NEE. The GPP subnetwork consists of three layers and estimates the ecosystem-level gross photosynthesis using a light-use efficiency (LUE) approach; in particular, instantaneous LUE is estimated by the first two layers, while GPP is calculated as the product between LUE and incoming shortwave radiation in the third layer. NN_C-part_ has a hybrid nature, and gross photosynthesis is partially constrained by emulating the LUE concept.

Each subnetwork relies on specific predictors. Distinguishing features of this model are that (a) GPP and R derived by other models are not used, (b) functional relationships are derived directly from the data, and (c) the network’s weights are tuned by training the machine learning only on NEE measurements. In this experiment we used the same predictors (VPD, incoming shortwave radiation, potential incoming radiation, *T*_a_, *T*_soil_, SWC, wind speed, and wind direction) and network structure as applied by Tramontana et al. (2020). However, to ensure the viability of this method, which is limited by the availability of both predictors and NEE measurements, we set lower requirements for the minimum percentage of measured data for both predictors and half hourly NEE. Moreover, data from all available years were pooled for use in a unique multi-year training process. In particular, we applied the following setting: for each year, less than 55% of predictors were gap-filled, and at least 365 half-hourly NEEs should be measured for both nighttime and daytime. Despite the high percentage of missing data in observations, gaps had generally short duration with limited effects on the uncertainty of predicted outputs. The final GPP_ANN_ products were derived by applying trained networks on meteorological inputs and thus do not include NEE data after network training. The modelled NEE from NN_C-part_ was compared with the measured NEE in Fig. B1b.

#### 2.3.3 GPP from COS flux measurements and an empirical LRU radiation relation

Based on previous soil chamber measurements at Hyytiälä forest it is known that the soil COS flux was −2.7 pmom^−2^s^−1^ on average with a variation of only 1pmol m^−2^s^−1^ during the growing season and a negligible diurnal variation (Kooijmans et al., 2017; Sun et al., 2018a). The average soil flux was thus first subtracted from the quality-filtered and gap-filled COS EC fluxes in order to derive the vegetation contribution to the ecosystem COS exchange. GPP was then calculated from the canopy COS fluxes (FCOS) using the formula (Sandoval-Soto et al., 2005; Blonquist et al., 2011)

(5) $${\text{GPP}}_{\text{COS}}=\frac{-\text{FCOS}}{\text{LRU}}\frac{{\left[{\text{CO}}_{2}\right]}_{\text{a}}}{{\left[\text{COS}\right]}_{\text{a}}},$$

where [CO_2_]_a_ and [COS]_a_ denote the atmospheric concentrations of CO_2_ and COS (in mol m^−3^), respectively, at the EC measurement height, measured by the QCL.

LRU was calculated as a function of PAR (LRUPAR) as described by the empirical equation of Kooijmans et al. (2019):

(6) $${\text{LRU}}_{\text{PAR}}=\frac{607.26}{\text{PAR}}+0.57$$

This LRU equation was derived from field chamber measurements (LRU_ch_) of pine branch CO_2_ and COS fluxes with two chambers placed at the top of the canopy in 2017 at the same site and thus being independent from the EC flux measurements (Kooijmans et al., 2019).

#### 2.3.4 GPP from COS flux measurements and LRU from stomatal optimization model

Finally, we estimated GPP from Eq. (5) using a new theoretical expression for LRU (LRU_CAP_) derived from the stomatal optimization model CAP (Dewar et al., 2018). Full details of the derivation are given in Appendix A. The LRU_CAP_ formulation was based on the following general expression for LRU given by Eqs. (10)–(11) of Wohlfahrt et al. (2012):

(7) $$\text{LRU}=\frac{1}{1-\frac{c_{\text{i}}}{c_{\text{a}}}}\frac{\frac{1}{1.21}+\frac{1}{1.14}\frac{g_{\text{s}}^{\text{COS}}}{g_{\text{b}}^{\text{COS}}}}{1+\frac{g_{\text{s}}^{\text{COS}}}{g_{\text{b}}^{\text{COS}}}+\frac{g_{\text{S}}^{\text{COS}}}{g_{\text{m}}^{\text{COS}}}},$$

where $g_{x}^{\text{COS}}(x=\text{b},\text{s},\text{m})$ are, respectively, the boundary layer, stomatal, and mesophyll conductances for COS, *c*_a_ is the atmospheric CO_2_ molar mixing ratio (molmol^−1^), *c*_i_ is the leaf intercellular CO_2_ molar mixing ratio (mol mol^−1^), and the numerical factors 1.21 and 1.14 are the ratios of the conductances of CO_2_ to COS for stomata and the boundary layer (Wohlfahrt et al., 2012). If it is assumed that the boundary layer and mesophyll conductances are infinite (as done by Dewar et al., 2018), Eq. (7) reduces to

(8) $$\text{LRU}=\frac{1}{1.21}{\left(1-\frac{c_{\text{i}}}{c_{\text{a}}}\right)}^{-1}.$$

An analytical expression for *c*_i_ was derived from the stomatal optimization model CAP by Dewar et al. (2018), according to which stomatal conductance maximize leaf photosynthesis, reflecting a trade-off between stomatal limitations to CO_2_ diffusion and non-stomatal limitations (NSLs) to carboxylation capacity. The CAP model predicts the value of *c*_i_ as an analytical function of various environmental and physiological factors. Inserting this function into Eq. (8), LRU_CAP_ can then be expressed as

(9) $${\text{LRU}}_{\text{CAP}}=\frac{1}{1.21}\frac{c_{\text{a}}}{c_{\text{a}}-{\text{Γ}}^{*}}\left(1+\sqrt{\frac{K_{\text{sl}}\left|\psi_{\text{c}}\right|}{1.6g_{\text{c}}\text{VPD}}}\sqrt{1+\frac{2{\text{Γ}}^{*}g_{\text{c}}}{\alpha \text{PAR}}}\right),$$

where Γ* is the CO_2_ photorespiratory compensation point (mol mol^−1^), *K*_sl_ the soil-to-leaf hydraulic conductance (mol m^−2^s^−1^MPa^−1^), *ψ*_c_ is the assumed critical leaf water potential (MPa) at which NSLs reduce photosynthesis to zero, gc is the carboxylation conductance in the absence of NSLs (mol m^−2^s^−1^) and *α* is the photosynthetic quantum yield (mol mol^−1^) in the absence of NSLs (Duursma et al., 2008; Dewar et al., 2018). While Γ*, and *α* vary seasonally with temperature, for simplicity we used fixed values representing the growing season averages of 50 × 10^−6^ and 0.05 mol mol^−1^, respectively (Bernacchi et al., 2001; Leverenz and Öquist, 1987; Mäkelä et al., 2008). In addition to PAR (mol m^−2^ s^−1^) and VPD measurements (mol mol^−1^), LRU_CAP_ requires soil moisture measurements through its dependence on the soil component of *K*_sl_. All parameter definitions and values are listed in Table 1.

LRU_CAP_ is based on a generic physiological model of stomatal function whose predictions have been successfully tested previously (e.g. Lintunen et al., 2020; Salmon et al., 2020; Dewar et al., 2021; Gimeno et al., 2019). The model parameters are all physiologically meaningful and can be measured independently or obtained from the literature. This formulation therefore represents a clear advance on previous COS-based methods based on empirical fitting (LRU_PAR_) because it provides a physiological explanation for variations in LRU that may be more robust when extrapolating to other sites.

In addition, LRU_CAP_ was calculated using a combination of literature values and fitted parameters by fitting the parameter combinations *X* = ļψ_c_ļ/(1.6*g_c_*) (MPam^2^s mol^−1^) and *Y* = 2Γ*g_c_/α(mol m^−2^s^−1^) to Eq. (9). This analysis was aimed at assessing the parameter sensitivity of LRU_CAP_. While the literature-based parameter values gave *X* = 2.5 and *Y* = 0.001, the fitting values were *X* = 2.64 and *Y* = 0.0033 and gave a slightly better agreement of LRU_CAP_ with LRU_ch_ (RMSE = 1.89, while without fitting RMSE = 2.01, Fig. B2). However, we emphasize that this fitting procedure was conducted purely in order to assess the model performance and is not a requirement for applying LRU_CAP_ in practice when literature-based parameter values are available. Moreover, the results presented in this article are not based on fitted parameter values but on literature values only.

## 3 Results and discussion

### 3.1 Environmental conditions

**Figure 1:**
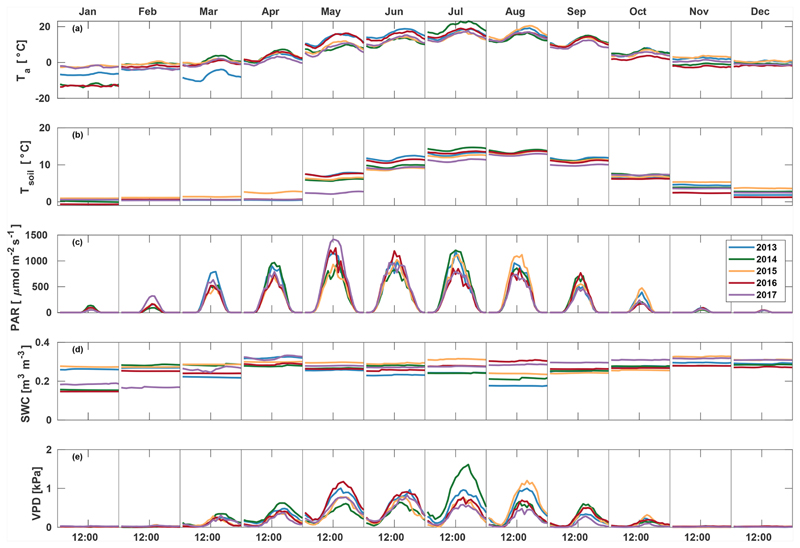
Median diurnal variation of *T*_a_, *T*_soil_, PAR, SWC, and VPD in different months during the measurement period 2013–2017.

March 2013 was colder than other years (average –7.0°C) and also had the highest average PAR (207.3 μmol m^−2^ s^−1^) and lowest soil moisture (0.23 m^3^m^−3^) (Fig. 1). A clear increase in VPD and decrease in soil moisture were seen in August 2013, with soil moisture decreasing from 0.24 in July to 0.19 m^3^m^−3^ in August and afternoon median VPD increasing to 1.00 kPa. July 2014 was warmer (19.0 °C) and dryer (VPD 0.88 kPa) than other years, but soil moisture remained high at 0.25 m^3^ m^−3^. In 2015, VPD increased from 0.44 in July to 0.62 kPa in August, and soil moisture decreased from 0.31 in July to 0.24m^3^ m^−3^ in August. May 2017 had high amounts of radiation (monthly average PAR of 478.4 μmol m^−2^ s^−1^), and soil temperature was low (3.4 °C), while soil moisture and VPD were at a normal level at 0.28 m^3^m^−3^ and 0.47 kPa, respectively. Soil moisture in September–December in 2017 was 10% higher than other years, while no significant differences between years were found in other environmental variables in late autumn.

### 3.2 GPP comparison from sub-daily to seasonal scales

**Figure 2:**
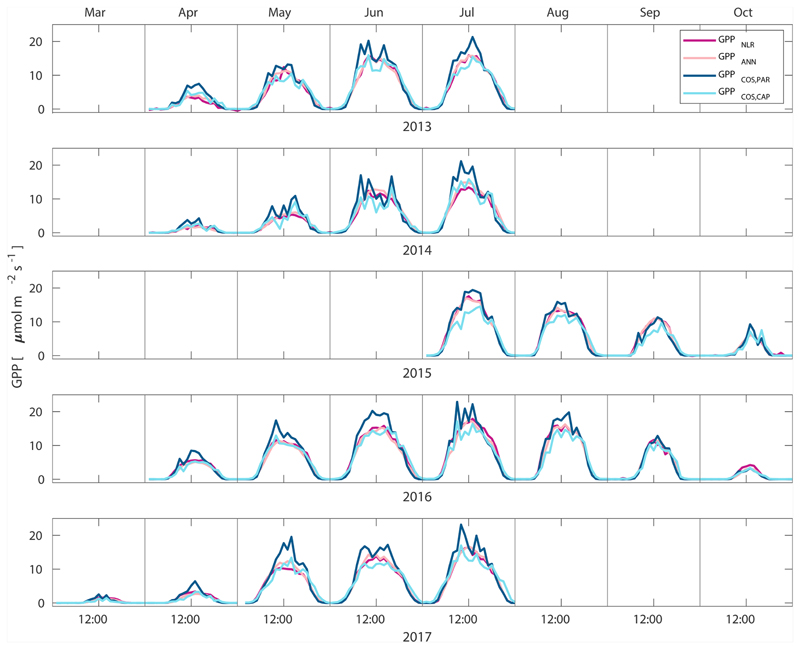
Median diurnal variation of GPP partitioned using a combined nighttime–daytime method (GPP_NLR_, purple line), GPP from artificial neural networks (GPP_ANN_, pink line), GPP from COS flux measurements with LRU determined according to Kooijmans et al. (2019) (GPP_COS,PAR_, dark blue line), and GPP from COS flux measurements using a new approach for LRU (Sect. 2.3.4, GPP_COS,CAP_,light blue line) in different months during the measurement period 2013–2017. Averaging was done to the same data points, and only months with more than 55% of data coverage were included.

**Figure 3:**
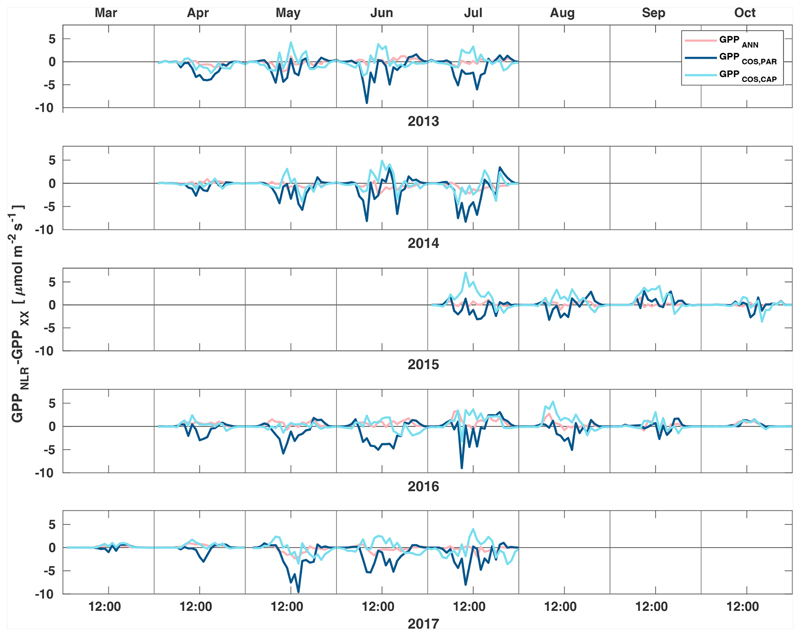
Diurnal variation of the difference of GPP_ANN_ (pink), GPP_COS,PAR_ (dark blue) and GPP_COS,CAP_ (light blue) to the reference GPP_NLR_ in different months during the measurement period 2013–2017. Averaging was done to the same data points and only months with more than 55% of data coverage were included.

**Figure 4:**
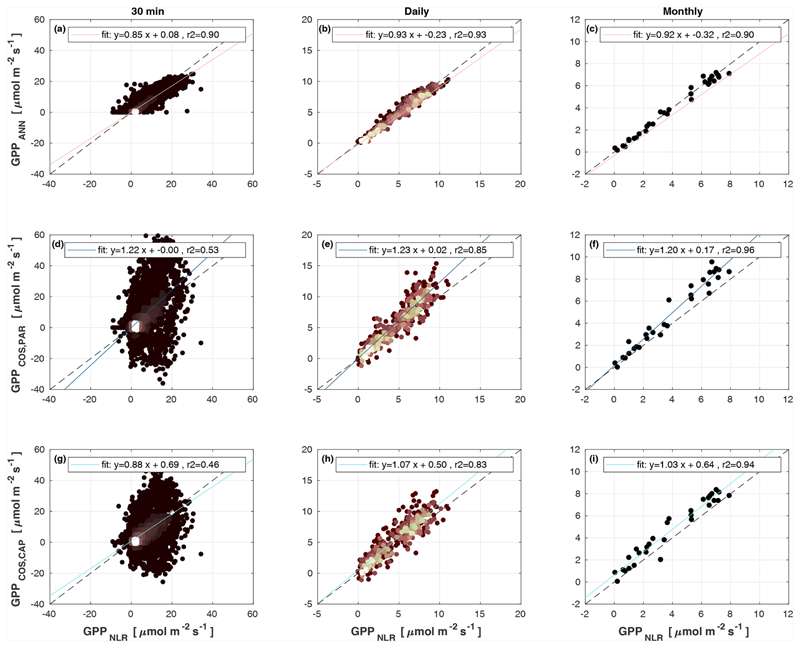
Scatter plots of GPP_ANN_, GPP_COS,PAR_, and GPP_COS,CAP_ against GPP_NLR_ in 30 min, daily, and monthly timescales. The colour of data points in 30 min and daily scatter plots indicates the data density, lighter colours indicating higher point density than dark ones.

**Figure 5:**
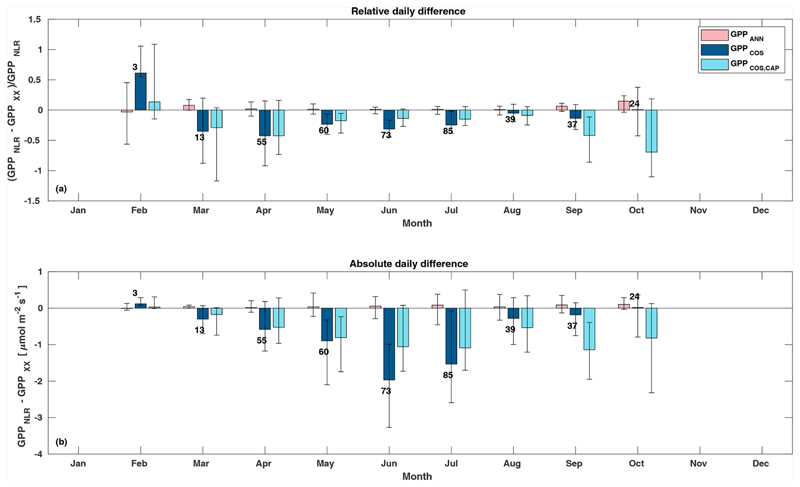
Relative **(a)** and absolute **(b)** difference of daily GPP_ANN_ (pink), GPP_COS,PAR_ (dark blue), and GPP_COS,CAP_ (light blue) to GPP_NLR_ in different months, averaged over the whole measurement period 2013–2017. Bars represent the median difference, and whiskers show the 25th and 75th percentiles. Numbers on top of the bars indicate how many daily flux data points have been used for calculating the medians. Median differences have been calculated using the same number of data points for each method in each month.

Midday GPP_ANN_ was on average 12 % higher than midday GPP_NLR_ during the summer months (May–July) in 2014 and 2017 (Figs. 2, 3, and 4a), opposite to the result found by Tramontana et al. (2020) in a comparison of GPP_ANN_ with standard FLUXNET partitioning during summer months at multiple sites. The difference between GPP_NLR_ and GPP_ANN_ during other months was negligible. We compared the more common use of air temperature as the respiration driver, GPPairT (instead of the average of soil and air temperatures), against GPP_NLR_ and found that the two methods agreed very well with each other at all timescales (Fig. B3). The small differences in the diurnal variations of GPP_NLR_ and GPP_ANN_ are thus not due to the choice of temperature measurement as respiration driver. During the measurement period 2013–2017, 30 min, daily, and monthly GPP_ANN_ did not differ statistically from GPP_NLR_ (tested with the ANOVA test; Figs. B4–B6). However, on 30 min timescale the GPP_ANN_ was on average 15 % lower than GPP_NLR_. The lower agreement of 30 min GPP_ANN_ and GPP_NLR_ than on longer timescales may have resulted from the NN_C-part_ method restricting GPP_ANN_ to only positive values, while GPP_NLR_ may take on negative values due to random noise in the NEE measurements. The relative and absolute differences of GPP_ANN_ to GPP_NLR_ are, however, very small when averaging over longer time periods (relative difference 2% on average during summer months, Fig. 5).

GPP_COS,PAR_ was very similar to GPP_NLR_ especially during morning and early evening (Figs. 2 and 3) but showed higher midday values than GPP_NLR_, especially during summer months (May–August) in all years. At the daily scale, GPP_COS,PAR_ was on average 23% higher than GPP_NLR_ (Figs. 4e and 5) and also differed from GPP_NLR_ and GPP_ANN_ statistically (*p* < 0.01) on 30 min and daily scales (ANOVA test). At monthly scale, there was no statistical difference to any of the other GPP methods.

**Figure 6:**
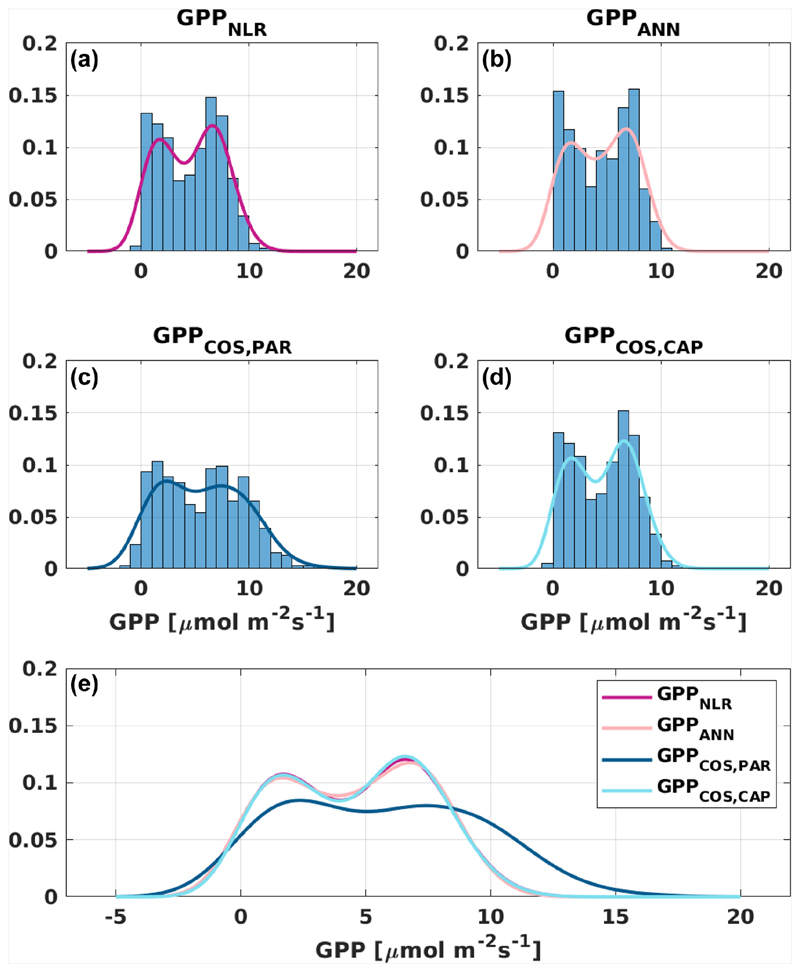
Distribution (bars) and probability density functions (lines) of daily average **(a)** GPP_NLR_, **(b)** GPP_ANN_, **(c)** GPP_COS,PAR_, and **(d)** GPP_COS,CAP_. All probability density functions are combined in **(e)** for better comparison.

Based on the CAP stomatal optimization model, LRU_CAP_ requires PAR, SWC, and VPD, as well as ecosystem-specific literature values, for some parameters as input variables. In contrast, LRU_PAR_ by Kooijmans et al. (2019) only uses PAR. LRU_CAP_ therefore takes into account additional effects of drought and air humidity on LRU. In spring, the diurnal variation of GPP_COS,CAP_ closely follows that of GPP_NLR_ and GPP_ANN_ until June (Figs. 2 and 3). Especially in June and July GPP_COS,CAP_ is lower than the other GPP estimates. At 30 min timescale GPP_COS,CAP_ is on average 12% lower than GPP_NLR_, but there is large scatter due to noisy FCOS measurements, like for GPP_COS,PAR_. However, there is less scatter in GPP_COS,CAP_ than GPP_COS,PAR_ (Fig. 4d and g), indicating that some of the scatter is due to LRU estimation. At daily scales GPP_COS,CAP_ is 7 % higher than GPP_NLR_, and at monthly scales the difference decreases to 3 %. However, there is no statistically significant difference between the 30 min and monthly values of GPP_NLR_ and GPP_COS,CAP_ (ANOVA test). The relative and absolute difference between GPP_COS,CAP_ and GPP_NLR_ is also generally smaller than between GPP_COS,PAR_ and GPP_NLR_ throughout the year (Fig. 5). In addition, GPP_COS,CAP_ reproduces the same two distinctive probability density function peaks as GPP_NLR_ and GPP_ANN_ at 1.7 and 6.6 μmol m^−2^ s^−1^, while GPP_COS,PAR_ finds weaker peaks at 2.4 and 7.4 μmol m^−2^ s^−1^ (Fig. 6). In summary, GPP_COS,CAP_ gives better agreement with traditional GPP_NLR_ partitioning than GPP_COS,PAR_. However, LRU_CAP_ was higher than LRU_ch_ and LRU_PAR_ at high radiation (PAR >1000 μmol m^−2^ s^−1^, Fig. B7a). This may reflect intrinsic differences in the dependence of LRU_PAR_ and LRU_CAP_ on environmental drivers (PAR, VPD, SWC) as both estimates of LRU are based on conditions at the top of the canopy.

LRU_CAP_ was also calculated based on a combination of literature values and the fitted parameters *X* and *Y* (Sects. 2.3.4 and A1) in order to assess the sensitivity to parameter values. While literature values gave *X* = 2.5 MPa m^2^ s mol^−1^ and *Y* = 0.001 mol m^−2^ s^−1^, fitting gave *X* = 2.64 MPa m^2^ s mol^−1^ and *Y* = 0.0033 mol m^−2^ s^−1^ and a slightly better agreement of LRU_CAP_ with measured LRU (RMSE = 1.89, while without fitting RMSE = 2.01). Thus, while *X* was close to its literature value, *Y* was estimated to be 3 times higher. This mismatch suggests there may be scope for further model improvement, such as the inclusion of dark respiration and/or finite mesophyll and boundary layer conductances in the LRU_CAP_ model. However, as the difference between fitted LRU_CAP_ and literature-based LRU_CAP_ (statistical significance tested with Student’s *t* test, *p* < 0.01) was not large, with a median difference of 4 %, and the applicability of the model without fitting is better, we decided to use the literature-based parameterization of LRU_CAP_ in this study, without fitting to LRU_ch_.

LRU_CAP_ was also calculated assuming finite mesophyll conductance as a further comparison (Sect. A2). The agreement of this method was better than assuming infinite mesophyll conductance at high PAR but worse at low PAR (Fig. B7d), very similar to the results from Maignan et al. (2021), who modelled LRU at Hyytiälä using the OR-CHIDEE model. This version of LRU_CAP_ was also fitted to measured LRU in terms of parameters *X* and *Y* (Sect. A2) to make the low PAR LRU_CAP_ better, which resulted in *X* = 3.45 and *Y* = 0.0057, both higher than their expected literature values. We thus concluded that the assumption of infinite, *g*m gives an estimate that is closest to LRU_ch_, although the assumption in itself is physiologically unrealistic. Kooijmans et al. (2019) found that internal conductance (a combination of mesophyll conductance and biochemical reactions) might limit leaf-scale FCOS during daytime. We find a better agreement of LRU_CAP_ with LRU_ch_ if *g*_m_ is assumed to be infinite, but there is a mismatch at high PAR, supporting the possibility that, *g*_m_ might indeed be a limiting factor under high radiation. In CAP, infinite or finite, *g*_m_ represents two contrasting hypotheses, in which NSLs act either entirely on photosynthetic capacity or entirely on, *g*_m_, respectively. In reality, NSLs may act on both photosynthetic capacity and, *g*_m_, with one or the other effect being dominant depending on environmental conditions. The contrasting abilities of each hypothesis to explain LRU_ch_ at low vs. high light might be explained by a shift in the action of NSLs from the photosynthetic capacity to, *g*_m_ as light increases. However, verifying this possibility lies beyond the scope of the present study.

We calculated the cumulative GPP estimates over May–July, 13 weeks around the peak growing season for each year (Table 2). Cumulative GPP_COS,PAR_ was on average 25% higher than cumulative GPP_NLR_ in all studied years. This is higher than the 4.3% difference reported in Spielmann et al. (2019) and 3.5% agreement reported in Commane et al. (2015). In contrast, cumulative GPP_COS,CAP_ varied from 17% higher in 2014 to 15% lower in 2015, and on average it was only 3% higher than cumulative GPP_NLR_. Cumulative GPP_ANN_ varied from 10% higher in 2014 to 9% lower in 2016 than GPP_NLR_, and on average it was 0.1% lower than GPP_NLR_. As stated above, overall GPP_ANN_ was closest to GPP_NLR_ out of the three other GPP estimates. GPP_COS,CAP_ was closer to both of the CO_2_-based GPP estimates than GPP_COS,PAR_. However, at high PAR, LRU_COS,CAP_ was higher than chamber-based measurements, leading to a lower GPP. Nevertheless, no firm conclusions can be drawn here as the LRU observations only cover measurements at the top of the canopy and may not reflect LRU over the whole canopy.

It has been suggested that, due to the Kok effect, leaf respiration is inhibited under radiation (Kok, 1949). This inhibition has been estimated to be approximately 13% in the evergreen needle–leaf forests during summer (Keenan et al., 2019). Measurements of CO_2_ isotope fluxes support the conclusion that, due to the Kok effect, GPP from traditional CO_2_ flux partitioning using the nighttime method is overestimated (Wehr et al., 2016). However, ecosystem respiration at the Hyytiälä forest site is dominated by soil respiration (Ilvesniemi et al., 2009) so that the Kok effect may be of limited importance in this ecosystem (Keenan et al., 2019; Yin et al., 2020). Reduced leaf respiration under radiation would be visible as a break point around the compensation point with a change in the slope of NEE against radiation. However, such a break point was not detected in our observations, as is demonstrated in Fig. B8. While it is possible that less radiated needles experience less inhibition than well radiated ones that cancel out at the ecosystem scale (Wohlfahrt et al., 2005), this test provides some insight into the problem. It is thus not expected that independent GPP estimates in Hyytiälä would necessarily result in lower GPP than the traditional methods. Moreover, Tramontana et al. (2020) showed that uncertainties and biases in NEE (and COS flux) measurements exceed those resulting from the possible Kok effect.

### 3.3 GPP responses to environmental conditions

**Figure 7:**
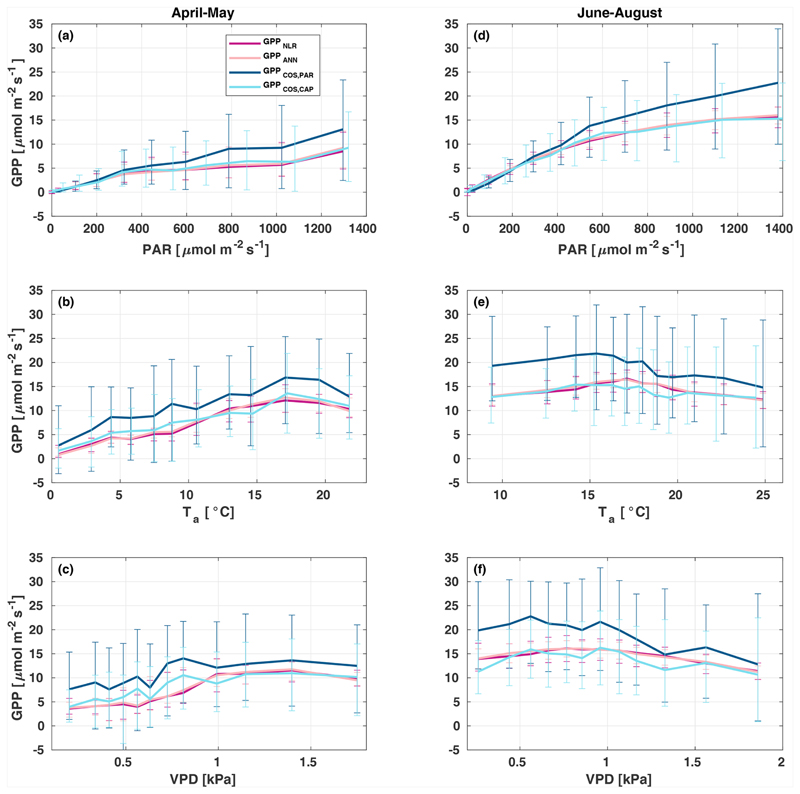
Responses of the different GPP estimates (GPP_NLR_ (purple), GPP_ANN_ (pink), GPP_COS,PAR_ (dark blue), and GPP_COS,CAP_, light blue) to environmental parameters – photosynthetically active radiation **(a, d)**, air temperature **(b, e)**, and vapour pressure deficit **(c, f)** – in spring **(a–c)** and summer **(d–f)**. Data are binned to 12 equally sized bins (same number of data points in each bin), and all GPPs have the same data coverage. Only measured (non-gap-filled) 30 min flux data were used, and GPP was filtered to include only PAR > 700 μmol m^2^ s^−1^ in responses to *T_a_* and VPD to avoid simultaneous correlation with PAR.

All four GPP estimates responded similarly to environmental forcing (PAR, *T*_a_, VPD) both in spring and summer (Fig. 7). In spring, all GPP estimates increased with increasing radiation levels, while in summer a saturation point was found at PAR > 500 μmol m^−2^ s^−1^ that could be linked to VPD limitation on stomatal conductance in the afternoon (Kooijmans et al., 2019). GPP_COS,PAR_ was higher than GPP_COs,CAp_ at PAR > 400 μmol m^−2^ s^−1^, while at low PAR values they agreed well with each other both in spring and summer, as well as with GPP_NLR_ and GPP_ANN_. GPP_COS,PAR_ thus has a stronger radiation response than the other GPP estimates due to a lower empirical LRU estimate than LRU_CAP_ at high PAR (Fig. B7). A similar PAR response was found in Spielmann et al. (2019), who studied GPP_COS,PAR_ with a traditional GPP partitioning method at four different sites in Europe. Although GPP_COS,CAP_ agrees well with both GPP_NLR_ and GPP_ANN_ at high PAR, it is likely underestimated due to high LRU_CAP_ at high PAR (Fig. B7).

In spring, increasing air temperature increased all GPP estimates similarly until *T*_a_ reached 17 °C. However, again GPP_COS,PAR_ was higher than other GPP estimates. In summer, air temperature did not have a notable effect on any GPP estimate. Responses to VPD were similar for each GPP estimates both in spring and summer. In spring, decreasing air humidity (increasing VPD) was associated with increased GPP until VPD > 0.7 kPa, after which VPD had little or no effect. The apparent increase in GPP with VPD in spring may be caused by the correlation of *T*_a_ with VPD, coinciding with the start of the growing season as the trees are not waterlimited after snowmelt. In summer, dryness started to limit GPP at VPD > 1 kPa. We found that similar to PAR and *T*_a_ responses, GPP_COS,PAR_ was higher than other GPP estimates at low VPD values but decreased to similar levels at high VPD (1 kPa) both in spring and summer. GPP_COS,PAR_ gives higher GPP at low VPD than the CO_2_-based methods, as does GPP_COS,CAP_ in spring (Fig. 7). This may indicate that some factor is limiting the photosynthesis reaction (e.g. biochemical limitations in CO_2_ assimilation) even though the diffusion into the leaf is not limited.

### 3.4 Uncertainties and limitations of the GPP methods

Because ANN fitting is purely based on the provided examples, GPP_ANN_ could be more sensitive to the uncertainty of (training) data with respect to the parametric partitioning methods. Moreover, it is sensitive to missing data especially in the case of long data gaps (Tramontana et al., 2020). The method also requires large data sets for training NN_C-part_, which may not be available at all measurement sites. However, GPP_ANN_ does not require prescribed relationships of GPP to environmental data, making it an attractive method for sites with good data availability.

GPP_COS,PAR_ uses an empirical PAR relation that is based on measurements at Hyytiälä forest. This PAR relation is site-specific and different compared to the one found by Yang et al. (2018). For this reason it is not known if and how it can be used at other sites, where it is suggested to retrieve it directly from observations. The choice of the empirical LRU–PAR relation at any given site is to some extent arbitrary. While the LRUPAR function is simple and thereby attractive, it does not take into account the different light conditions inside the canopy, stomatal regulation during drought, or the effects of non-stomatal limitations on photosynthesis. Moreover, being an empirical model, it does not provide a process-based understanding for LRU. While the results of GPP_COS,PAR_ are promising, we found a 25% difference in midday GPP during summer, similar to that found by Kooijmans et al. (2019). We did not find as good an agreement with CO_2_-based GPP estimates as Asaf et al. (2013), who found an agreement within 15% using a constant LRU of 1.6 in Mediterranean pine forests and crop fields. However, they also reported higher GPP_COS_ assumed to be related to soil COS uptake, which was not measured or taken into account in their GPP calculations. In our study, we subtracted an average measured soil flux (Sun et al., 2018a) from the ecosystem COS uptake. As the diurnal variation in soil COS exchange was small (less than 1 pmol m^−2^ s^−1^) throughout the season, averaging did not make a large difference, and thus soil does not explain the differences found here. However, as soil COS flux measurements are not necessarily available at all sites, this may be one source of uncertainty in wider applications. Yang et al. (2018) studied COS flux components and GPP_COS_ in a Mediterranean citrus orchard and found GPP_COS_ to be on average 7% lower than traditionally partitioned GPP. They also presented a light-dependent and seasonally varying LRU which, however, could not be applied to Hyytiälä COS fluxes due to the very different ecosystem types studied, indicating that the PAR responses may differ between ecosystems.

GPP_COS,CAP_ may be more applicable at other sites than GPP_COS,PAR_ because it is based on a generic physiological model of stomatal behaviour, which requires only literature-based parameter values and simple meteorological variables as inputs. However, as for LRU_PAR_, LRU_CAP_ should also be tested at other sites against measured LRU to verify its applicability to other ecosystems. The version of LRU_CAP_ assuming infinite mesophyll conductance, while giving reasonable results in comparison with LRU_c_h, is clearly physiologically unrealistic. The formulation of LRU_CAP_ with finite gm did not compare as well with LRU_ch_ at Hyytiälä forest (RMSE = 2.58, median difference to LRU_ch_ 22 %), especially during low light conditions, but may compare better at other measurement sites.

One source of uncertainty in GPP estimates based on LRU_CAP_ and LRUPAR is that both LRU predictions are calculated from radiation measurements at the top of the canopy, where there is no shading by foliage, although the theoretical dependence of LRU_CAP_ on radiation is more generally applicable throughout the canopy. The branch chamber measurements (on which the empirical LRUPAR function is based) were also made at the top of the canopy. The measured needles were thus well-adjusted to high radiation conditions. Therefore, we did not take into account light penetration and scattering through the canopy. However, the needles and leaves within the canopy are also well adjusted to low light conditions and may be more efficient with their stomatal control in varying light conditions than needles on top of the canopy. Thus, this may not be a large source of uncertainty. However, it is also possible that LRU varies throughout the canopy due to different light conditions.

## 4 Conclusions

Daily GPP_ANN_ and GPP_NLR_ did not differ significantly, and differences were also small at sub-daily and seasonal timescales. GPP_COS,PAR_ was higher than GPP_NLR_ at all timescales studied, including the estimate of 3-month cumulative GPP during the peak growing season. In contrast, GPP_COS,CAP_, a new method based on stomatal optimization theory, gave better agreement with GPP_NLR_ at all timescales and was also less scattered than GPP_COS,PAR_ at a 30 min timescale.

The LRU_CAP_ function provides a new theoretical underpinning for COS-based GPP estimates that can be used at other measurement sites, potentially without requiring additional branch chamber measurements. LRU_CAP_ represents a significant improvement on previous LRU functions based on site-specific empirical regressions. However, LRU_CAP_ overestimated LRU at high radiation when compared to LRU observations at the top of the canopy, leading to a lower midday GPP_COS,CAP_, especially in summer. This discrepancy may result from the assumption of infinite mesophyll conductance, or the absence of dark respiration, in the underlying stomatal optimization model. LRU_CAP_ would benefit from further testing at other measurement sites with COS and CO_2_ branch flux measurements, including measurements inside the canopy for better canopy-integrated LRU estimates.

Although COS flux measurements are noisier, more expensive, and more difficult than those of CO_2_, they provide an opportunity for better process-based understanding of photosynthesis in comparison to more traditional CO_2_-based estimates of GPP. In addition to COS, other proxies such as solar-induced fluorescence and isotopic flux measurements should be tested simultaneously to properly investigate their deficiencies and advantages in estimating GPP and processes underlying photosynthesis.

The establishment of large long-term ecosystem research infrastructures (e.g. ICOS, NEON, TERN; see Papale, 2020) – involving sites equipped with eddy covariance systems that could potentially also host COS, SIF (solar-induced fluorescence), and isotope sensors – together with the planned launch of the FLEX satellite in 2025 (https://earth.esa.int/eogateway/missions/flex, last access: 28 June 2022) that will provide global vegetation fluorescence measurements, opens up a new phase in monitoring and understanding plant photosynthesis. Our results also underline the important role of small-scale ecophysiological measurements and models in underpinning these larger-scale initiatives.

## Supplementary Material

Appendix

## Figures and Tables

**Table 1:** Explanations, literature values, and sources of the parameters used in the LRU_CAP_ formulation for Hyytiälä forest. *c*_a_was derived from the measurements in Kooijmans et al. (2019), and SWC, PAR, and VPD from measurements done in this study. Soil-related values (*K*_soil,sat_, *r*_cyl_, SWC_sat_, and *b*) are for soil horizon B (which was considered to be representative of the rooting zone), where the SWC measurements were also made.

Symbol	Definition	Default value or formula and unit	Source
*c*_a_	Atmospheric CO_2_molar mixing ratio	415 × 10^−6^ mol mol^−1^	Measured
Γ*	Photorespiratory compensation point of CO_2_	50 × 10^−6^ mol mol^−1^	Bernacchi et al. (2001)
*g*_c_	Carboxylation conductance in the absence of non-stomatal limitations	0.5 mol m^−2^s^−1^	Dewar et al. (2018)
*ψ*_c_	Critical leaf water potential	–2 MPa	Dewar et al. (2018)
*α*	Photosynthetic quantum yield	0.05 mol mol^−1^	Leverenz and Öquist (1987)
*K*_sl_	Leaf-specific soil-to-leaf hydraulic conductance	$\frac{K_{\text{soil }}K_{\text{X}}}{K_{\text{soil }}+K_{\text{X}}};{\text{molm}}^{-2}{\text{s}}^{-1}{\text{MPa}}^{-1}$	
*K*_X_	Leaf-specific root-to-leaf xylem hydraulic conductance	0.78 × 10^−3^mol m^−2^s^−1^MPa^−1^	Duursma et al. (2008)
*K*_soil_	Leaf-specific soil hydraulic conductance	$\frac{R_{1}}{\text{LAI}}\frac{2\pi k_{\text{soil }}}{\log\left(\frac{r_{\text{cyl }}}{r_{\text{root }}}\right)};{\text{molm}}^{-2}{\text{s}}^{-1}{\text{MPa}}^{-1}$	
*k*_soil_	Soil hydraulic conductivity	$k_{\text{soil,sat }}{\left(\frac{\text{SWC}}{{\text{SWC}}_{\text{sat}}}\right)}^{2b+3};{\text{molm}}^{-1}{\text{s}}^{-1}{\text{MPa}}^{-1}$	
*k*_soil,sat_	Saturated soil hydraulic conductivity	5.7 mol m^−1^s^−1^MPa^−1^	Duursma et al. (2008)
*R*_1_	Root length index	5300 m^−1^	Nikinmaa et al. (2013)
LAI	Leaf area index, all-sided	8 m^2^m^−2^	Measured
*r*_cyl_	Radius of the cylinder of soil accessible to a root	0.00458 m	Duursma et al. (2008)
*r*_root_	Fine root radius	0.3 × 10^−3^ m	Nikinmaa et al. (2013)
SWC_sat_	Saturation soil water content	0.52 m^3^m^−3^	Duursma et al. (2008)
SWC	Soil water content	m^3^ m^−3^	Measured
*b*	Parameter of the soil water retention curve	4.46	Duursma et al. (2008)
VPD	Vapour pressure deficit	mol mol^−1^	Measured
PAR	Photosynthetically active radiation	mol m^−2^s^−1^	Measured

**Table 2:** Cumulative GPP (gCm^−2^) over May-July with different GPP estimates. All sums are calculated from the same data coverage, and the fraction of gap-filled flux data (CO_2_ flux for GPP_NLR_, COS flux for GPP_COS,PAR_ and GPP_COS,CAP_) is presented in parentheses. GPP_ANN_ does not include gap-filled NEE data since it is based on meteorological variables. * In 2015, the cumulative sum covers only July.

Year	GPP_NLR_	GPP_ANN_	GPP_COS,PAR_	GPP_COS,CAP_
2013	481 (0.16)	473	597 (0.28)	510 (0.28)
2014	294 (0.20)	324	414 (0.24)	343 (0.24)
2015	193 (0.23)*	188^*^	212(0.31)*	165 (0.31)^*^
2016	623 (0.40)	565	722 (0.43)	599 (0.43)
2017	387 (0.35)	399	522 (0.34)	428 (0.34)

## Data Availability

The flux data and all GPP estimates used in this study are available from https://doi.org/10.5281/zenodo.6940750 (Kohonen et al., 2022). Environmental data used in the study are available from http://urn.fi/urn:nbn:fi:att:a8e81c0e-2838-4df4-9589-74a4240138f8 (Aalto et al., 2019). The most recent version of the data is available from https://smear.avaa.csc.fi (last access: 9 June 2020).
