# Supplementary material for: Intercomparison of methods to estimate gross primary production based on CO2 and COS flux measurements
Source: Biogeosciences. Author manuscript; Available in PMC 2022 Sep 27. (PMC7613647; doi:10.5194/bg-19-4067-2022)
Supplement: Appendix [file EMS154471-supplement-Appendix.pdf]

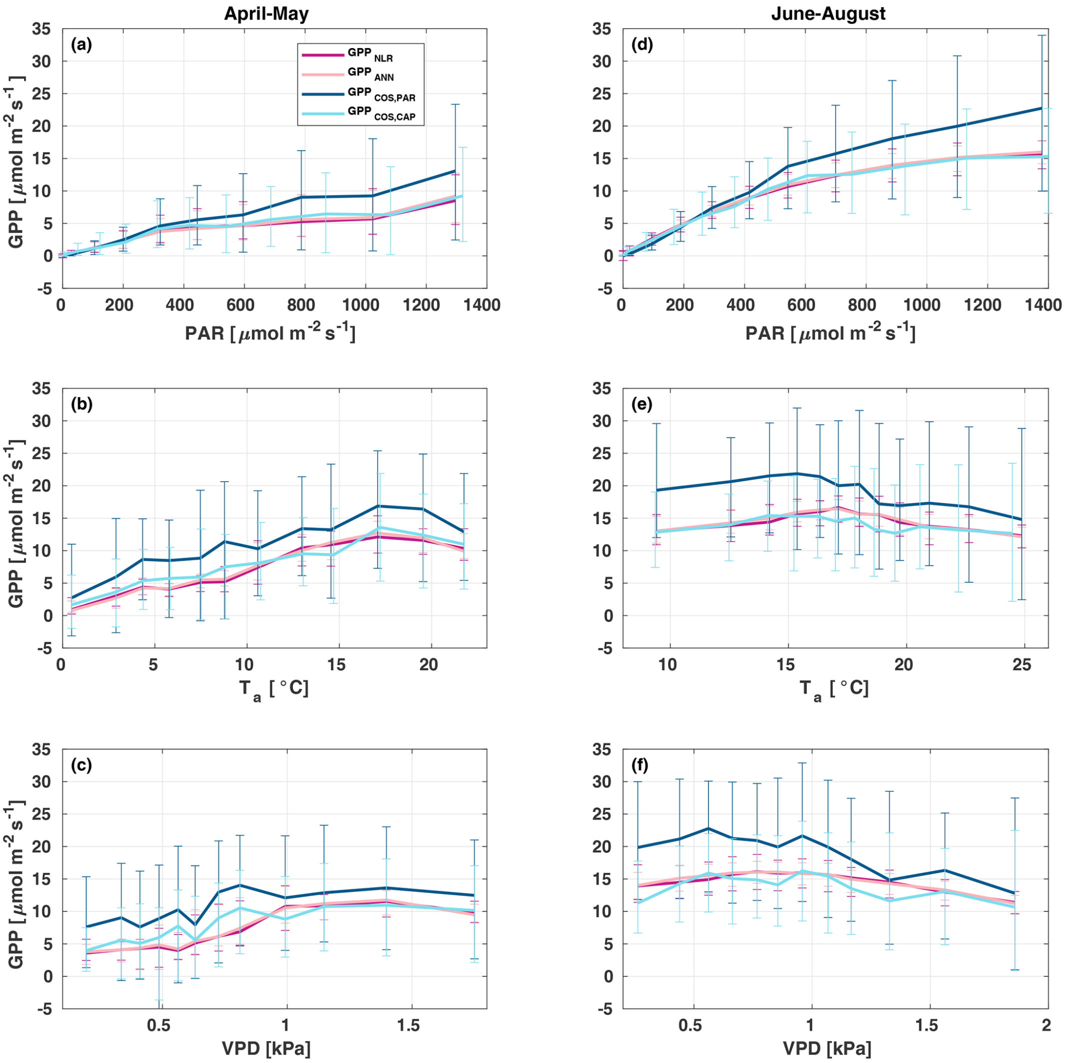

Figure 7. Responses of the different GPP estimates (GPP_{NLR} (purple), GPP_{ANN} (pink), $GPP_{COS,PAR}$ (dark blue), and $GPP_{COS,CAP}$, light blue) to environmental parameters – photosynthetically active radiation **(a, d)**, air temperature **(b, e)**, and vapour pressure deficit **(c, f)** – in spring **(a–c)** and summer **(d–f)**. Data are binned to 12 equally sized bins (same number of data points in each bin), and all GPPs have the same data coverage. Only measured (non-gap-filled) 30 min flux data were used, and GPP was filtered to include only PAR > 700 μmol $m^2 s^{-1}$ in responses to T_a and VPD to avoid simultaneous correlation with PAR.

rescence), and isotope sensors – together with the planned launch of the FLEX satellite in 2025 (https://earth.esa.int/eogateway/missions/flex, last access: 28 June 2022) that will provide global vegetation fluorescence measurements, opens up a new phase in monitoring and understanding plant photosynthesis. Our results also underline the important role of small-scale ecophysiological measurements and models in underpinning these larger-scale initiatives.

Appendix A: LRU predicted by the CAP stomatal optimization model

A1 LRU_{CAP} assuming infinite mesophyll conductance

The general expression for the leaf relative uptake ratio (LRU) derived from the diffusion laws for COS and CO_2 (Wohlfahrt et al., 2012) is

$$\mathrm{LRU} = \frac{1}{1-\frac{c_i}{c_a}} \frac{\frac{1}{1.21} + \frac{1}{1.14}\frac{g_s^{COS}}{g_b^{COS}}}{1+\frac{g_s^{COS}}{g_b^{COS}}+\frac{g_s^{COS}}{g_m^{COS}}}, \quad \text{(A1)}$$

where $g_x^{COS} (x = \mathrm{b, s, m})$ are the boundary layer, stomatal and mesophyll conductances for COS, respectively, c_a and

c_i are the atmospheric and leaf intercellular CO_2 molar mixing ratios ($mol\,mol^{-1}$), respectively, and the numerical factors 1.21 and 1.14 are the ratios of the conductances of CO_2 to COS for stomata and the boundary layer, respectively.

If it is assumed that boundary layer and mesophyll conductances are infinite, Eq. (A1) reduces to

$$\mathrm{LRU} = \frac{1}{1.21}\left(1 - \frac{c_i}{c_a}\right)^{-1}. \tag{A2}$$

We derived c_i from the CAP stomatal optimization model (Dewar et al., 2018), according to which stomatal conductance adjusts to maximize the rate of leaf photosynthesis (A) through a trade-off between stomatal and non-stomatal limitations. Our photosynthesis model is based on that of Thornley and Johnson (1990) (their Eq. 9.12i), modified to include non-stomatal limitations (NSLs):

$$A = \left(1 - \frac{\psi_{\mathrm{leaf}}}{\psi_c}\right)\frac{\alpha Q g_c (c_i - \Gamma^*)}{\alpha Q + g_c (c_i + \Gamma^*)}, \tag{A3}$$

where α is the photosynthetic quantum yield ($mol\,mol^{-1}$) in the absence of NSLs, Q ($mol\,m^{-2}\,s^{-1}$) is photosynthetically active radiation (PAR), g_c ($mol\,m^{-2}\,s^{-1}$) is the initial slope of the A–c_i response curve in the absence of NSLs, Γ^* ($mol\,mol^{-1}$) is the photorespiratory CO_2 compensation point, ψ_{leaf} (MPa) is the leaf water potential, and ψ_c (MPa) is the critical leaf water potential at which NSLs reduce photosynthesis to zero. In Eq. (A3), NSLs are represented as an apparent downregulation of the $A - c_i$ response curve by a factor that decreases with decreasing leaf water potential, as has been observed in numerous experiments (e.g. Lintunen et al., 2020; Salmon et al., 2020). Consequently, as stomatal conductance increases there is a trade-off between increased CO_2 supply and increased NSLs such that A has a maximum at some optimal value of stomatal conductance.

We used Eq. (A3) rather than the Farquhar photosynthesis model (Farquhar et al., 1980) because, in the latter, the abrupt switch from RuBisCo to electron transport limitation introduces artificial discontinuities in the CAP solution for optimal stomatal conductance (Dewar et al., 2018), whereas in Eq. (A3) there is a smooth transition from CO_2 limitation to light limitation, and no such discontinuities occur. The parameter g_c is equivalent to $V_{\mathrm{cmax}}/(k_m + \Gamma^*)$ in the Farquhar model.

The CAP solution for optimal stomatal conductance (Dewar et al., 2018) predicts that

$$\frac{c_i - \Gamma^*}{c_a - \Gamma^*} = \frac{1}{1+\beta}, \tag{A4}$$

where

$$\beta = \sqrt{\frac{1.6D}{K_{\mathrm{sl}}|\psi_c|}\left(\frac{1}{g_c} + \frac{2\Gamma^*}{\alpha Q}\right)^{-1}}, \tag{A5}$$

in which D is vapour pressure deficit (VPD; $mol\,mol^{-1}$), and K_{sl} is the leaf-specific soil-to-leaf hydraulic conductance ($mol\,m^{-2}\,s^{-1}\,MPa^{-1}$). Writing Eq. (A2) in the equivalent form

$$\mathrm{LRU} = \frac{1}{1.21}\frac{c_a}{c_a - \Gamma^*}\left(1 - \frac{c_i - \Gamma^*}{c_a - \Gamma^*}\right)^{-1} \tag{A6}$$

and substituting the CAP prediction from Eqs. (A4) and (A5) then give

$$\mathrm{LRU_{CAP}} = \frac{1}{1.21}\frac{c_a}{c_a - \Gamma^*}\left(1 + \sqrt{\frac{K_{\mathrm{sl}}|\psi_c|}{1.6 D g_c}}\sqrt{1 + \frac{2\Gamma^* g_c}{\alpha Q}}\right). \tag{A7}$$

In Eq. (A7) all the parameters are physiologically meaningful and can be measured independently or obtained from the literature because the underlying CAP model is based entirely on such parameters. This contrasts with the use of the stomatal optimization model of Medlyn et al. (2011), for example, which contains an undetermined parameter (λ, interpreted as the marginal water cost of carbon gain) that must be empirically fitted.

Nevertheless, to assess the performance of $\mathrm{LRU_{CAP}}$ obtained from literature-based parameter values, we compared it with $\mathrm{LRU_{CAP}}$ obtained by fitting the two key parameter combinations $X = |\psi_c|/(1.6 g_c)$ and $Y = 2\Gamma^* g_c/\alpha$, in terms of which Eq. (A7) may be written as

$$\mathrm{LRU_{CAP}} = \frac{1}{1.21}\frac{c_a}{c_a - \Gamma^*}\left(1 + \sqrt{\frac{K_{\mathrm{sl}}X}{D}}\sqrt{1 + \frac{Y}{Q}}\right). \tag{A8}$$

Parameters X and Y were optimized to minimize the RMSE of $\log(\mathrm{LRU_{CAP}})$ to measured $\log(\mathrm{LRU})$, due to the logarithmic nature of LRU, with MATLAB's *fminsearch* function. However, we emphasize that this fitting procedure was conducted purely in order to assess the model performance and is not a requirement for applying $\mathrm{LRU_{CAP}}$ in practice when literature-based parameter values are available. Moreover, the results presented in this study are not based on the optimized values but on literature values only.

A2 $\mathrm{LRU_{CAP}}$ assuming finite mesophyll conductance

In the case that mesophyll conductance is not assumed to be infinite (but boundary layer conductance is infinite), Eq. (A1) becomes

$$\mathrm{LRU} = \frac{1}{1.21}\frac{1}{1 + \frac{g_s^{\mathrm{COS}}}{g_m^{\mathrm{COS}}}}\left(1 - \frac{c_i}{c_a}\right)^{-1}. \tag{A9}$$

If we further assume that the ratios of stomatal to mesophyll conductances are the same for CO_2 and COS, then from $g_s^{CO_2}(c_a - c_i) = g_m^{CO_2}(c_i - c_c)$, where c_c is the chloroplast CO_2 molar mixing ratio ($mol\,mol^{-1}$), we can make the substitution

$$\frac{g_s^{\mathrm{COS}}}{g_m^{\mathrm{COS}}} = \frac{g_s^{CO_2}}{g_m^{CO_2}} = \frac{c_i - c_c}{c_a - c_i} \tag{A10}$$

in Eq. (A9) to obtain

$$\mathrm{LRU} = \frac{1}{1.21}\left(1 - \frac{c_c}{c_a}\right)^{-1}, \tag{A11}$$

which reduces to Eq. (A2) when mesophyll conductance is infinite (since then $c_c = c_i$). As noted above, CAP represents NSLs in terms of an apparent downregulation of the $A - c_i$ response curve (Eq. A3). This empirical observation may be interpreted in various ways: as a downregulation of photosynthetic efficiencies (α and g_c) in the chloroplast, a downregulation of mesophyll conductance ($g_m^{CO_2}$), or some combination of the two. In the case when NSLs act entirely on g_m^{COS} with no effect on the biochemical efficiencies, A is given as a function of the chloroplast CO_2 concentration by (cf. Eq. A3)

$$A = \frac{\alpha Q g_c (c_c - \Gamma^*)}{\alpha Q + g_c (c_c + \Gamma^*)}. \tag{A12}$$

In this case, since Eq. (A3) still holds, we obtain the same optimal CAP solution for stomatal conductance and c_i (Eq. A4) as before but now with an additional prediction for the finite (but variable) mesophyll conductance as implied by Eq. (A12), which links the chloroplast CO_2 concentration (c_c) to the CAP solution of A. From Eq. (A12),

$$c_c - \Gamma^* = \frac{\left(\frac{\alpha Q}{g_c} + 2\Gamma^*\right) A}{\alpha Q - A}. \tag{A13}$$

The CAP solution for stomatal conductance is given by (Dewar et al., 2018)

$$g_s = \frac{\alpha Q}{\frac{\alpha Q}{g_c} + 2\Gamma^*} \frac{x\theta}{x\beta^2 + (1-x)(xw+1)}, \tag{A14}$$

where

$$\theta = 1 - \frac{\psi_{soil}}{\psi_c}, \tag{A15}$$

$$w = \frac{c_a - \Gamma^*}{\frac{\alpha Q}{g_c} + 2\Gamma^*}, \tag{A16}$$

$$x = \frac{c_i - \Gamma^*}{c_a - \Gamma^*} = \frac{1}{1+\beta}, \tag{A17}$$

in which ψ_{soil} (MPa) is the soil water potential. Substituting x as a function of β into Eq. (A14) and simplifying give

$$g_s = \frac{\alpha Q}{\frac{\alpha Q}{g_c} + 2\Gamma^*} \frac{\theta}{\beta\left(1 + \beta + \frac{w}{1+\beta}\right)}. \tag{A18}$$

We then find the CAP solution for A as follows:

$$\begin{aligned} A &= g_s (c_a - c_i) \\ &= g_s (c_a - \Gamma^*)(1 - x) \\ &= g_s (c_a - \Gamma^*) \frac{\beta}{1+\beta} \\ &= \frac{\alpha Q (c_a - \Gamma^*)}{\frac{\alpha Q}{g_c} + 2\Gamma^*} \frac{\theta}{(1+\beta)^2 + w}. \end{aligned} \tag{A19}$$

Substituting this into Eq. (A13) and simplifying then give

$$\begin{aligned} \frac{c_c - \Gamma^*}{c_a - \Gamma^*} &= \frac{\theta}{(1+\beta)^2 + (1-\theta) w} \\ &= \frac{\theta}{(1+\beta)^2 + (1-\theta)\frac{g_c (c_a - \Gamma^*)}{\alpha Q + 2\Gamma^* g_c}}, \end{aligned} \tag{A20}$$

which can be combined with Eq. (A11) to give the solution of $\mathrm{LRU_{CAP}}$ with finite mesophyll conductance.

As for $\mathrm{LRU_{CAP}}$ with infinite mesophyll conductance, we also fitted this version with respect to parameters X and Y in order to compare it with the performance of the model using literature-based values. For this procedure, β and w were expressed in terms of X and Y,

$$\beta = \frac{1}{\sqrt{\frac{K_{sl} X}{D}\left(1 + \frac{Y}{Q}\right)}} \tag{A21}$$

and

$$w = \frac{c_a - \Gamma^*}{2\Gamma^*} \frac{1}{\frac{Q}{Y} + 1}, \tag{A22}$$

and then substituted into Eq. (A20). However, as for the infinite g_m solution, this fitting procedure was conducted purely in order to assess the model performance and is not a requirement for applying $\mathrm{LRU_{CAP}}$ in practice when literature-based parameter values are available.

Appendix B: Additional figures

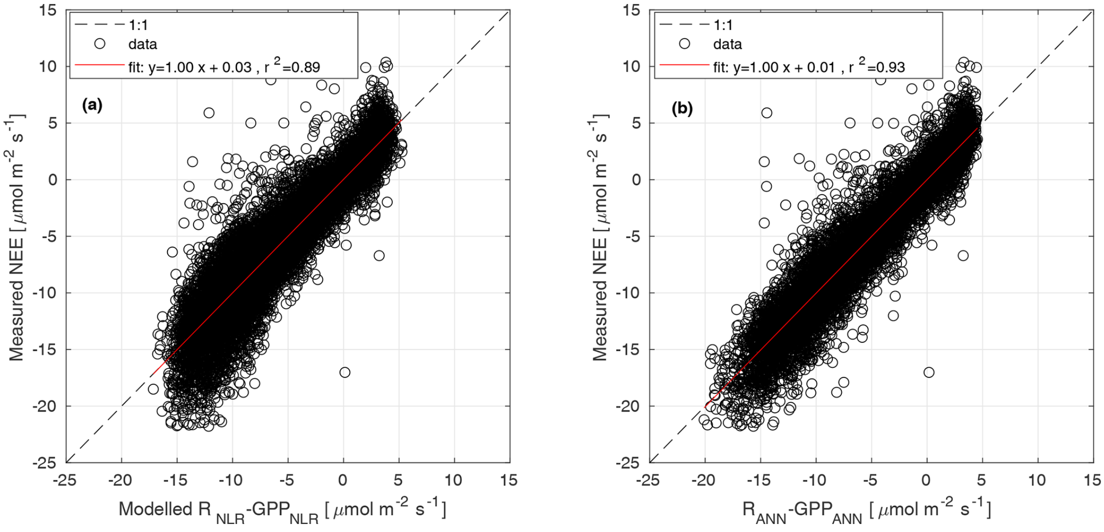

Figure B1. Modelled against measured NEE using **(a)** NLR and **(b)** ANN models for modelling NEE.

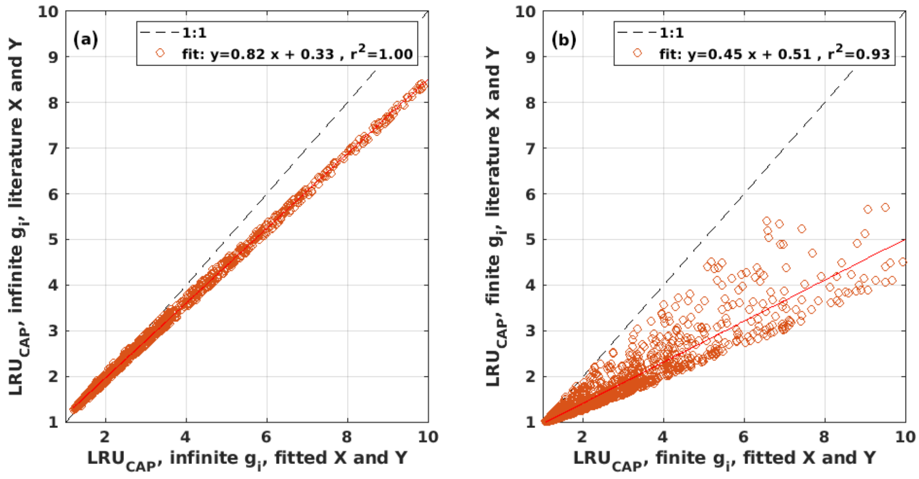

Figure B2. Scatter plots of LRU_{CAP} using the literature values against LRU_{CAP} using the optimized parameter values when assuming **(a)** infinite or **(b)** finite mesophyll conductance.

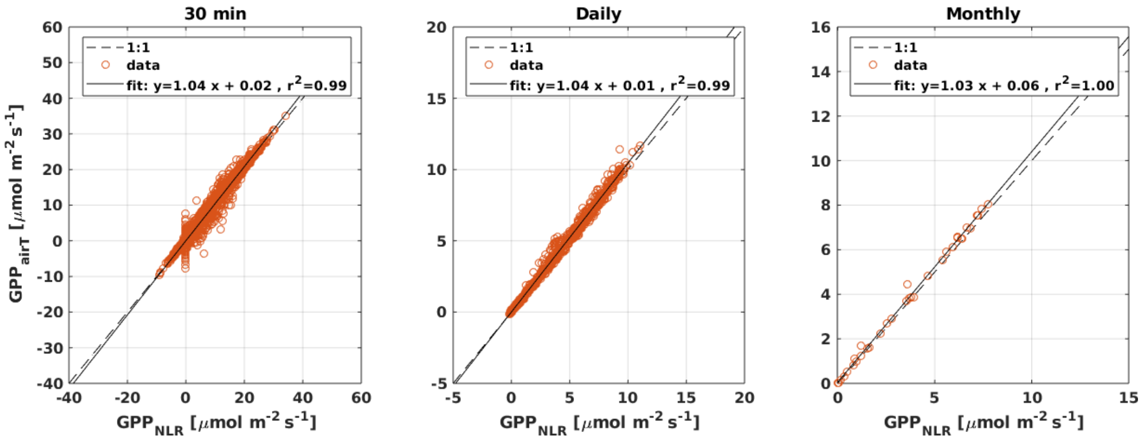

Figure B3. Scatter plots of GPP_{airT} that uses only air temperature as the driver for respiration against GPP_{NLR} that uses an average of air and soil temperatures as the respiration driver at 30 min, daily, and monthly timescales. The solid black line is the least-squares linear fit to the data.

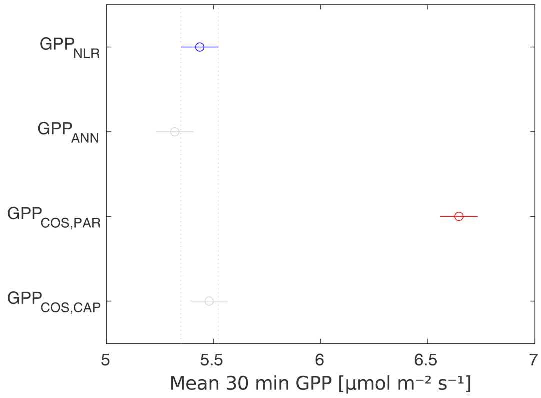

Figure B4. ANOVA test results for 30 min GPP data. Gray bars indicate no difference to the reference (blue), and red bars indicate statistical difference to the reference. The results show that only $GPP_{COS,PAR}$ differs statistically from GPP_{NLR} at 30 min timescale.

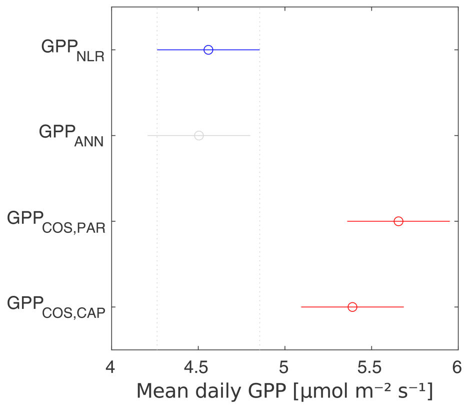

Figure B5. ANOVA test results for daily GPP data. Gray bars indicate no difference to the reference (blue), and red bars indicate statistical difference to the reference. The results show that both $GPP_{COS,PAR}$ and $GPP_{COS,CAP}$ differ statistically from both GPP_{NLR} and GPP_{ANN} at daily scale. $GPP_{COS,PAR}$ and $GPP_{COS,CAP}$ do not differ from each other.

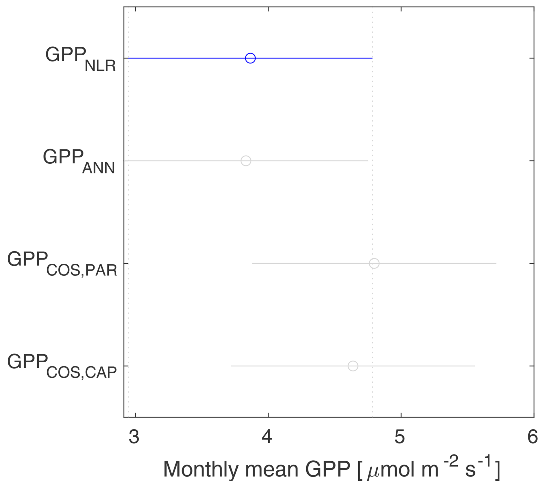

Figure B6. ANOVA test results for monthly GPP data. Gray bars indicate no difference to the reference (blue), and red bars indicate statistical difference to the reference. The results show that all GPPs are statistically the same at monthly scale.

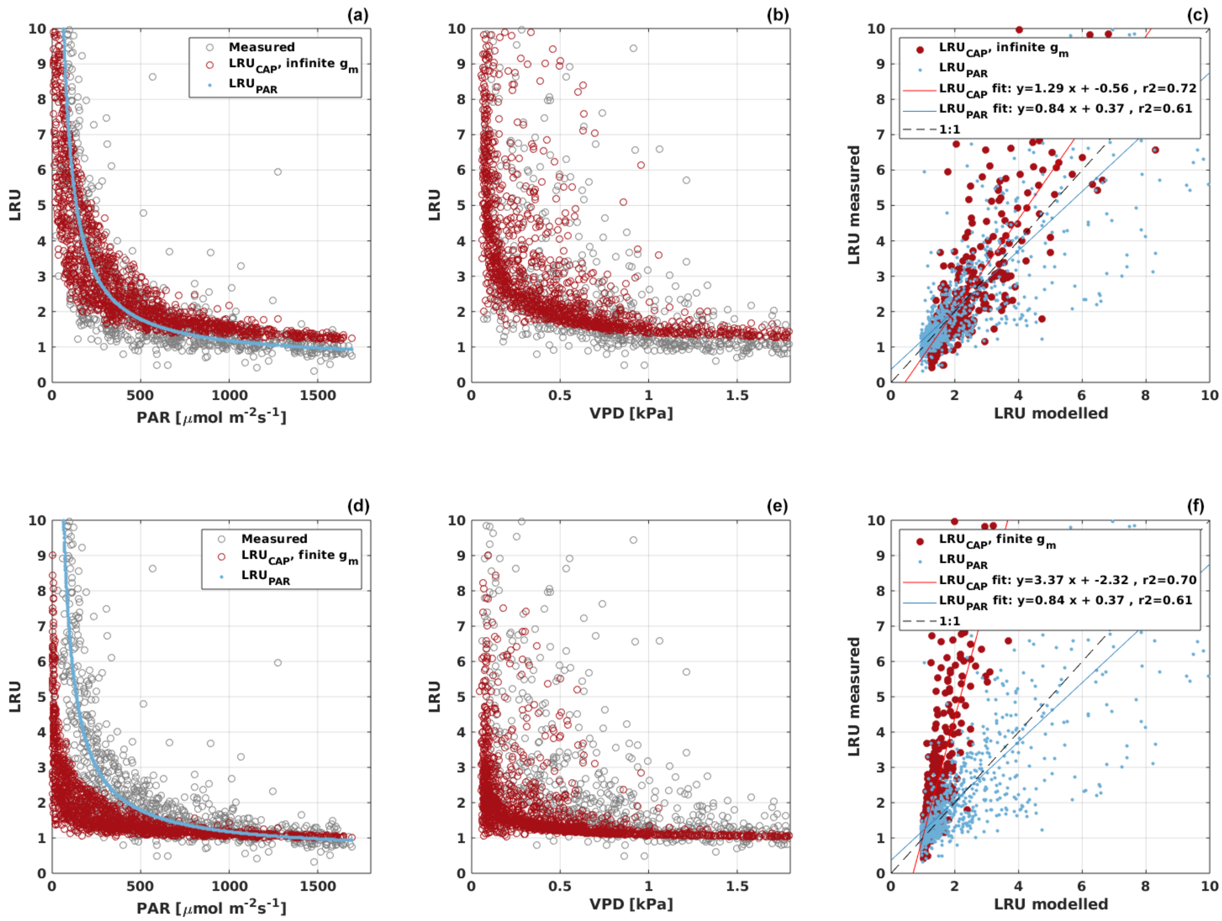

Figure B7. LRU derived from chamber measurements (gray) and modelled LRU_{PAR} (blue) and LRU_{CAP} (red) assuming infinite **(a–c)** or finite **(d–f)** mesophyll conductance (g_m) in LRU_{CAP} against PAR and VPD. Subplots **(c)** and **(d)** compare the chamber-measured LRU against modelled LRU_{PAR} and LRU_{CAP}.

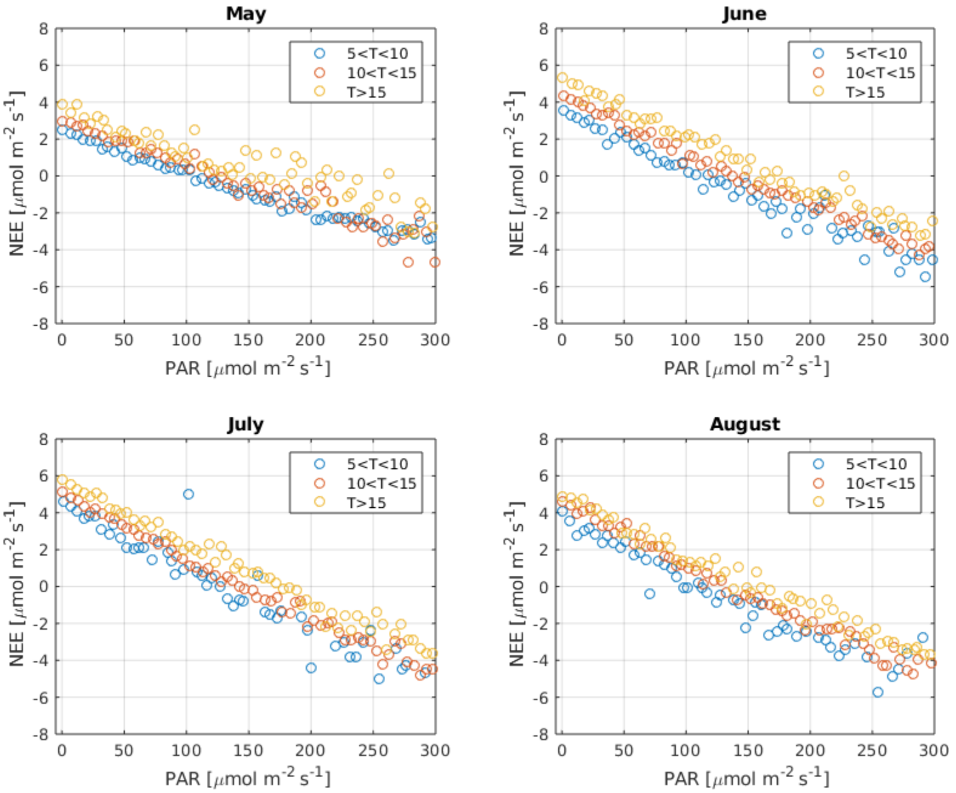

Figure B8. Net ecosystem exchange (NEE) against photosynthetically active radiation (PAR) close to the compensation point during May, June, July, and August. Data are binned to different air temperature classes: 5 °C < T_a < 10 °C (blue), 10 °C < T_a < 15 °C (orange), and T_a > 15 °C (yellow).

Data availability. The flux data and all GPP estimates used in this study are available from https://doi.org/10.5281/zenodo.6940750 (Kohonen et al., 2022). Environmental data used in the study are available from http://urn.fi/urn:nbn:fi:att:a8e81c0e-2838-4df4-9589-74a4240138f8 (Aalto et al., 2019). The most recent version of the data is available from https://smear.avaa.csc.fi (last access: 9 June 2020).

Author contributions. KMK, IM, and TV designed the study. KMK, PK, and LMJK performed the measurements and flux processing. RD, AM, and KMK developed the new LRU formulation. GT provided the GPP estimate by artificial neural networks. DP gave insight into all GPP method uncertainties and study design and commented on the manuscript. All authors contributed by commenting on the study design, results, and the manuscript. KMK wrote the manuscript with contributions from all co-authors.

Competing interests. The contact author has declared that none of the authors has any competing interests.

Disclaimer. Publisher's note: Copernicus Publications remains neutral with regard to jurisdictional claims in published maps and institutional affiliations.

Acknowledgements. Special thanks to Helmi Keskinen, Sirpa Rantanen, Janne Levula, and other Hyytiälä technical staff for all their support with the measurements.

Financial support. This research has been supported by the Academy of Finland (grant nos. 118780, 312571, 282842, 337549, and 342930), the H2020 European Research Council (grant nos. 755617 and 742798), the H2020 Environment (grant no. 820852), and ICOS Finland (grant no. 3119871). Specifically, Gianluca Tramontana was supported by the European Research Council (ERC) under the ERC-2017-STG SENTIFLEX project (grant no. 755617), Linda M. J. Kooijmans was supported by the ERC advanced funding scheme (AdG 2016, grant number 742798, project abbreviation COS-OCS), and Dario Papale was supported by the E-SHAPE H2020 project (grant no. 820852) and the ICOS-ETC. Kukka-Maaria Kohonen was supported by the Vilho, Yrjö, and Kalle Väisälä foundation. Aleksanteri Mauranen was supported by the Doctoral Programme in Atmospheric Sciences of the University of Helsinki.
